# A Bioactive Compound-Loaded Zinc-Aminoclay Encapsulated, Pickering Emulsion System for Treating Acne-Inducing Microbes

**DOI:** 10.3390/ijms24119669

**Published:** 2023-06-02

**Authors:** Seong-Hyeon Kim, In-Sun Bae, Hyun Uk Lee, Ju-Young Moon, Young-Chul Lee

**Affiliations:** 1 Department of BioNano Technology, Gachon University, 1342 Seongnam-daero, Sujeong-gu, Seongnam-si 13120, Republic of Korea; shaera@gachon.ac.kr; 2 Swsonaki Inc., Gwangyang Frontier-Valley 3rd, 30 Gaseok-ro, Incheon 22827, Republic of Korea; swsonaki@naver.com; 3 Research Center for Materials Analysis, Korea Basic Science Institute, Daejeon 34133, Republic of Korea; leeho@kbsi.re.kr; 4 Department of Beauty Design Management, Hansung University, 116, Samseongyo-ro 16gil, Seoul 02876, Republic of Korea

**Keywords:** Pickering emulsion, acne, antimicrobial, extended release, zinc-aminoclay (ZnAC), microwave-assisted *Opuntia humifusa* (MA-OHE)

## Abstract

Acne is a common skin condition caused by the growth of certain bacteria. Many plant extracts have been investigated for their potential to combat acne-inducing microbes, and one such plant extract is microwave-assisted *Opuntia humifusa* extract (MA-OHE). The MA-OHE was loaded onto zinc-aminoclay (ZnAC) and encapsulated in a Pickering emulsion system (MA-OHE/ZnAC PE) to evaluate its therapeutic potential against acne-inducing microbes. Dynamic light scattering and scanning electron microscopy were used to characterize MA-OHE/ZnAC PE with a mean particle diameter of 353.97 nm and a PDI of 0.629. The antimicrobial effect of MA-OHE/ZnAC was evaluated against *Staphylococcus aureus* (*S. aureus*) and *Cutibacterium acnes* (*C. acnes*), which contribute to acne inflammation. The antibacterial activity of MA-OHE/ZnAC was 0.1 and 0.025 mg/mL to *S. aureus* and *C. acnes*, respectively, which were close to naturally derived antibiotics. Additionally, the cytotoxicity of MA-OHE, ZnAC, and MA-OHE/ZnAC was tested, and the results showed that they had no cytotoxic effects on cultured human keratinocytes in a range of 10–100 μg/mL. Thus, MA-OHE/ZnAC is suggested to be a promising antimicrobial agent for treating acne-inducing microbes, while MA-OHE/ZnAC PE is a potentially advantageous dermal delivery system.

## 1. Introduction

Human skin provides a fertile environment for specific Gram-positive microbes, including *Cutibacterium acnes* (*C. acnes*) and *Staphylococcus aureus* (*S. aureus*), which are associated with inflammatory acne. Various studies have attempted to treat acne-inducing microbes using the antibiotic properties of antioxidant plant extracts (Table 1). In particular, microwave-assisted *Opuntia humifusa* (*O. humifusa*) extract (MA-OHE) has been found to have antimicrobial activity and is useful for treating acne-inducing microbes [1,2,3]. In addition to antimicrobial activity, *O. humifusa* extract exhibits beneficial effects, especially in preventing cancer, diabetes, and cardiovascular disease in humans, through its various bioactive compounds [4,5,6]. The microwave-assisted extraction method was conducted due to its high polyphenol yield and shorter extraction duration [7,8,9]. The radiation from the microwave heats the inside of the cell and breaks the cell wall, allowing the bioactive compounds to dissolve into the extraction solvent [10].

Combining the advantages of microwave-assisted extraction and Zinc-aminoclay (ZnAC), this approach is expected to show promising results in combating acne-inducing microbes. ZnAC, also known as 3-aminopropyl zinc phyllosilicate, is an aminopropyl functionalized 2D layered material formed by the sol-gel transition of 3-aminopropyltriethoxysilane (APTES) and cationic zinc ions [28]. During the synthesis process, APTES attaches to the surface of the layered silicate, forming covalent bonds between the silicate and the zinc ions [29]. Significantly, ZnAC exhibits strong antimicrobial effects but low toxicity [30]. The aminopropyl groups of ZnAC can be delaminated to a cationic state when dispersed in water, allowing negatively charged drug molecules to be easily loaded and exhibit antimicrobial effects [31,32]. As a result of this property, like other two-dimensional (2D) materials, aminoclays can be used to load drug molecules through hydrogen bonding or covalent bonding [32,33,34].

Because ZnAC nanoparticles were dispersed in water, a solidified encapsulation system is essential for stability [31]. An emulsion system stabilized by solid lipid particles, also called a Pickering emulsion, is substantially more stable than other classical emulsion systems [35]. The major advantages of the Pickering emulsion system include high resistance to coalescence and the ability to undergo emulsification [36]. Pickering emulsions have a wide range of potential applications in biotechnology [37]. For drug delivery, Pickering emulsions can provide controlled release of drugs and improve their bioavailability [38,39]. In the food industry, they can be used to encapsulate flavorings or nutrients, and in cosmetics, they can be used to stabilize emulsions and enhance the skin-penetrating ability of active ingredients [40,41,42]. In this study, bioactive compound-loaded ZnAC (MA-OHE/ZnAC) was encapsulated using a Pickering emulsion system, and its antimicrobial efficacy was evaluated.

## 2. Results and Discussion

### 2.1. Preparation of MA-OHE/ZnAC

**Figure 1:**
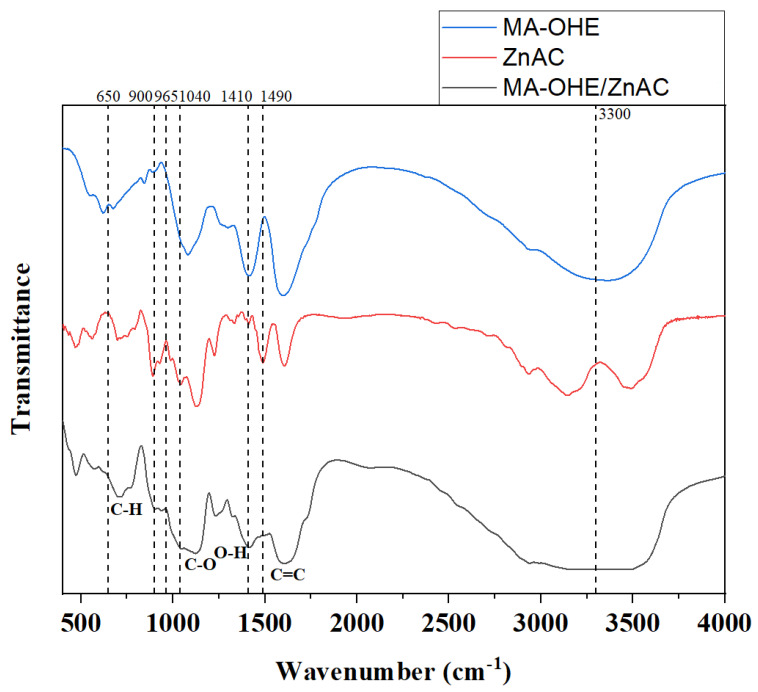
FT-IR spectra of MA-OHE, ZnAC, and MA-OHE/ZnAC. Labels indicate shifted peaks of MA-OHE/ZnAC.

FT-IR spectra showing information related to the functional groups and peak alteration of MA-OHE, ZnAC, and MA-OHE/ZnAC are shown (Figure 1). The C-H aromatic ring at the 710 cm^−1^ region, C-O tertiary alcohol at the 1124–1205 cm^−1^ region, O-H carboxylic acid at the 1395–1440 cm^−1^ region, and C=C α,β-unsaturated ketone at the 1620–1610 cm^−1^ region were identified in MA-OHE/ZnAC. Peak shifts of MA-OHE/ZnAC were noted at 650, 900, 965, 1040, 1410, and 1490 cm^−1^, respectively. In addition, the N-H and OH stretching bands for the MA-OHE/ZnAC overlapped and displayed a combined vibrational band in the wavelength range 3500–3000 cm^−1^. Similar to other 2D nanomaterials, peak shifting and overlapping were attributed to the bioactive compound loaded on the ZnAC surface and occurred mainly due to covalent bonding [29,33]. In this experiment, the covalent bonds between MA-OHE and ZnAC could be explained by newly detected polar bonds.

### 2.2. Morphological Structure of MA-OHE/ZnAC PE

**Figure 2:**
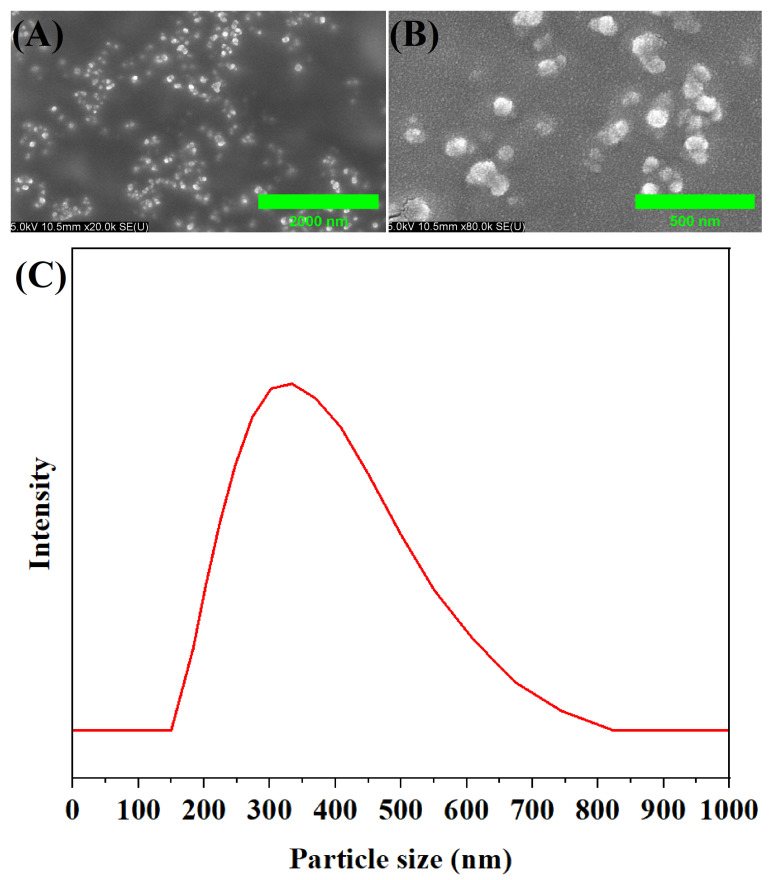
SEM image of MA-OHE/ZnAC PE at ×20.0 K magnification (**A**); SEM image of MA-OHE/ZnAC PE at ×80.0 K magnification (**B**); particle size distribution of MA-OHE/ZnAC PE (**C**).

SEM images revealed the morphological structure of MA-OHE/ZnAC PE (Figure 2A,B). Homogeneously dispersed particles were observed at 20.0 K magnification, and submicron-sized particles were observed at 80.0 K magnification, respectively. The particle size distribution highlighted the presence of submicron-sized particles in MA-OHE/ZnAC PE (Figure 2C). The particle size distribution curve of MA-OHE/ZnAC suggested that the particle size is between 200 and 600 nm (Z-average), with a calculated mean particle diameter of 353.97 nm and a PDI of 0.629. In this study, nanosized particles were found to facilitate rapid and safe dermal delivery, resulting in enhanced penetration and absorption of active ingredients through the skin barrier [43]. The spherical submicron-sized particles observed in the Pickering emulsion were comparable to those reported in the literature [44,45].

### 2.3. Bioactive Compound Entrapment Efficiency

The entrapment efficiency of MA-OHE/ZnAC and MA-OHE/ZnAC PE was 39.40 ± 1.06% and 87.08 ± 3.10%, respectively, and the R^2^ value of the standard curve was >0.99 (Figure S1A,B). Bioactive compound entrapment by MA-OHE/ZnAC and high entrapment efficiency of MA-OHE/ZnAC PE were demonstrated; similar entrapment efficiencies of Pickering emulsions were reported earlier [46,47]. The chemical bonding of bioactive compounds to ZnAC is believed to contribute to the high entrapment efficiency of MA-OHE/ZnAC PE. However, the increased entrapment of MA-OHE/ZnAC PE is considered to be attributed to the presence of free MA-OHE entrapped within the MA-OHE/ZnAC PE. The current research related to MA OHE/ZnAC PE is reinforcing its superiority with the emerging interest in lipid nanoparticles.

### 2.4. In Vitro Bioactive Compound Dissolution Test

**Figure 3:**
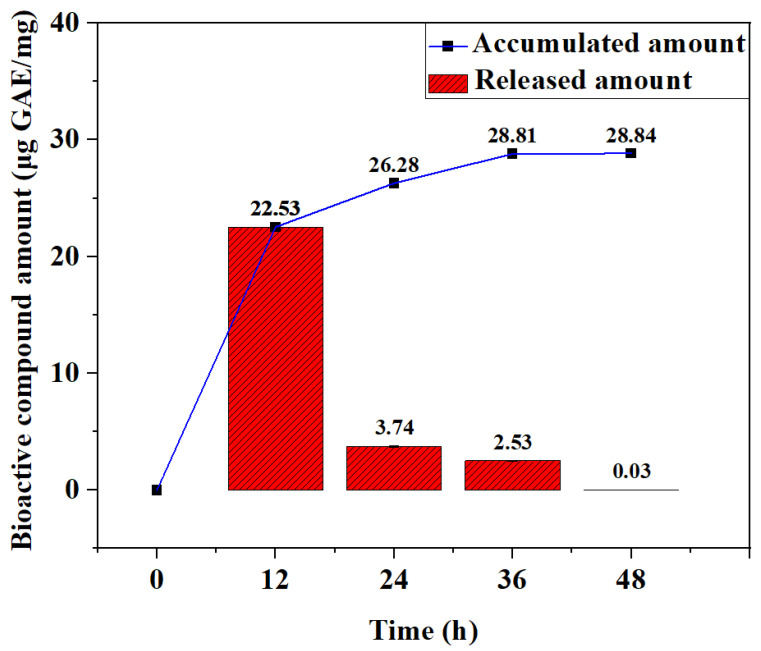
Dissolution behavior of MA-OHE/ZnAC (n = 3, *p* < 0.05).

The amount of released phenolic compound was expressed as micrograms of gallic acid equivalents per milligram (μg GAE/mg). The total phenolic amount of the MA-OHE/ZnAC was 39.40 ± 1.06%, as stated earlier, and the R^2^ value of the gallic acid standard curve was >0.999 (Figure S2). The dissolution characteristic of MA-OHE/ZnAC legends extended the bioactive compound release characteristic of MA-OHE/ZnAC (Figure 3). A total of 22.53 μg GAE/mg (57.18% of the total phenolic amount of MA-OHE/ZnAC) was released in the first 12 h. Subsequently, phenolic compound equivalent to 6.28 μg GAE/mg (15.94% of the total phenolic amount of MA-OHE/ZnAC) was released until 36 h and that equivalent to 0.03 μg GAE/mg of phenolic compound was released between 36 and 48 h, indicating the completion of release. The enhanced antimicrobial susceptibility property of MA-OHE/ZnAC against *C. acnes* could be attributed to the extended bioactive compound release property of the MA-OHE/ZnAC.

### 2.5. In Vitro Drug Permeation Kinetics

**Figure 4:**
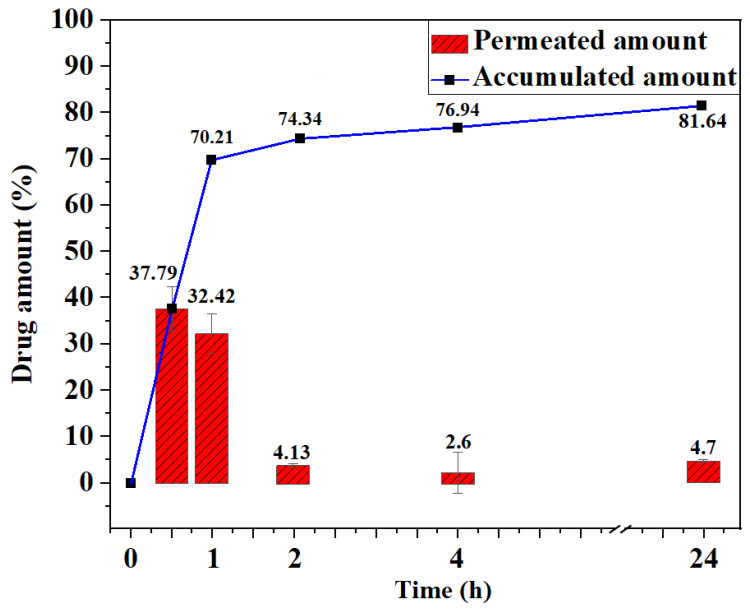
In vitro drug permeation kinetics of MA-OHE/ZnAC PE (n = 3, *p* > 0.1).

In vitro drug permeation kinetic analysis revealed rapid Fickian behavior of MA-OHE/ZnAC PE with a 5.12 × 10^−9^ m^2^/s diffusion coefficient and −4.17 × 10^−9^ flux (Figure 4 and Figure S3); 70.21% of the drug permeated in the first 1 h without any lag time. Subsequently, only 11.43% of MA-OHE/ZnAC PE permeated over 23 h, with a total of 81.64% of the drug being permeated. The rapid permeation was thought to be attributed to the submicron-sized particles of MA-OHE/ZnAC PE. The MA-OHE/ZnAC PE exhibits rapid permeation behavior and extended release, making it a unique drug delivery system.

### 2.6. Antimicrobial Susceptibility Test

MIC values of MA-OHE, ZnAC, and MA-OHE/ZnAC were determined using the broth micro-dilution method (Table 2). MIC values of MA-OHE for *S. aureus* and *C. acnes* were 0.75 mg/mL and 7.5 mg/mL, respectively. ZnAC showed a higher antimicrobial activity for *S. aureus* and *C. acnes*, with MIC values of 0.1 mg/mL and 0.05 mg/mL, respectively. The MIC values of MA-OHE/ZnAC to *S. aureus* and *C. acnes* were 0.1 mg/mL and 0.025 mg/mL, respectively. MIC values of ZnAC and MA-OHE/ZnAC were close to the MIC values of naturally derived antibiotics, suggesting their strong antimicrobial activity [11,14]. However, the antimicrobial effect of MA-OHE/ZnAC on *C. acnes* was higher than that of ZnAC and was believed to be related to the extended bioactive compound release property of the MA-OHE/ZnAC.

### 2.7. In Vitro Cell Viability Assay

**Figure 5:**
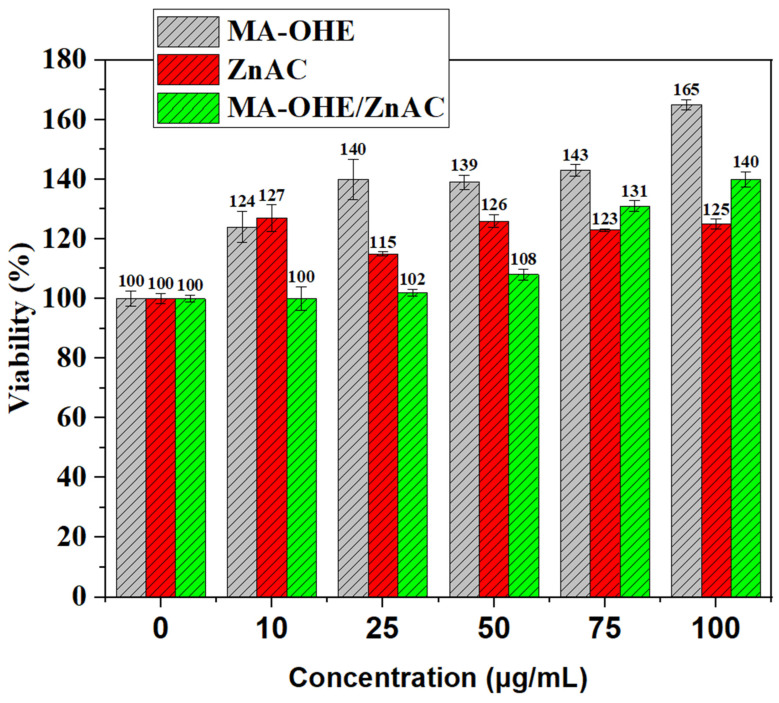
HaCaT cell viability in response to ZnAC, MA-OHE, and MA-OHE/ZnAC exposure (n = 3, *p* < 0.1) Values are expressed as mean ± standard deviation.

MA-OHE, ZnAC, and MA-OHE/ZnAC had no cytotoxic effects on HaCaT cells as determined using the WST assay (Figure 5). Indeed, MA-OHE increased the viability of the HaCaT cells in a dose-dependent manner when used in the range of 10–100 μg/mL, except at 50 μg/mL. MA-OHE/ZnAC and ZnAC increased the cell viability in a non-dose-dependent manner. These results were comparable to those reported in the literature [6,31]. Hence, collectively, our results provide evidence that MA-OHE/ZnAC enhances the proliferation of human keratinocytes instead of cytotoxic effects in the range of 10–100 μg/mL.

## 3. Materials and Methods

### 3.1. Materials and Reagents

Fresh *O. humifusa* cladodes, from plants grown for 3 years, were collected from Uiryeong-gun, Gyeongsangnam-do, Korea. Zinc chloride (ZnCl_2_) and ethanol were purchased from Daejung Chemicals & Metals Co. Ltd. (Siheung-si, Gyeonggi-do, Republic of Korea). APTES was purchased from Tokyo Chemical Industry (Nihonbashi-honcho, Chuo-ku, Tokyo, Japan). Folin-Denis reagent, sodium carbonate (Na_2_CO_3_), glycerol, and cetyl palmitate were purchased from Sigma-Aldrich (St. Louis, MO, USA). Phosphate buffered saline (PBS) was purchased from Thermo Fisher Scientific Inc. (Waltham, MA, USA). A Milli-Q Millipore filter system (Millipore Co., Billerica, MA, USA) was used to obtain distilled (DI) water. Potassium bromide (KBr) was procured from PIKE Technologies (Fitchburg, WI, USA).

### 3.2. Formulation of MA-OHE Loaded Emulsion

#### 3.2.1. Microwave-Assisted Extraction of *O. humifusa* (MA-OHE)

The microwave-assisted extraction method was used to extract *O. humifusa* [10]. Briefly, fresh *O. humifusa* cladodes were washed with DI water and dried in ambient air. The dried cladodes were then mixed with an equal weight of DI water and blended using a domestic blender to obtain the *O. humifusa* mixture. Next, 30 g of the *O. humifusa* mixture was irradiated using a domestic microwave oven (Magic MMO-20M7, SK magic, Jongno-gu, Seoul, Republic of Korea) at 1000 W for 8 min. The irradiated *O. humifusa* mixture was ground using a pestle, to which 30 mL of DI water was added, and the diluted mixture was passed through a 0.22 µm pore-sized hydrophilic filter (Sartorius, Göttingen, Germany) to remove impurities and debris. Finally, the filtrate was freeze-dried under conditions of −45 °C and 10 Pa to obtain MA-OHE.

#### 3.2.2. Synthesis of ZnAC and MA-OHE/ZnAC

ZnAC was synthesized using a sol-gel transition process previously reported [48]. Briefly, 8.4 g of ZnCl_2_ was dispersed in 200 mL of ethanol and stirred gently for 10 min. Next, 10 mL of APTES was added and stirred gently for 16 h. The mixture was then centrifuged at 4000 rpm for 20 min, and the supernatant was discarded. The precipitate was dried in a dry oven at 60 °C, and the dried ZnAC was ground using a pestle to form ZnAC powder.

For the aqueous phase, 40 mg of ZnAC and the same weight of MA-OHE powder were mixed with 20 mL of DI water and gently stirred for 6 h at ambient conditions. Bioactive compound-loaded ZnAC (MA-OHE/ZnAC) was obtained by centrifugation at 4000 rpm for 20 min and further analyzed. Fourier transform-infrared (FT-IR) spectroscopy (FT/IR-4100 spectrometer, Jasco, Easton, MD, USA) was performed to determine the functional groups of MA-OHE, ZnAC, and MA-OHE/ZnAC. The KBr pellet was measured as a baseline and scanned from 4000 to 400 cm^−1^, and H_2_O and CO_2_ signals were removed after data acquisition.

#### 3.2.3. Preparation of MA-OHE/ZnAC PE

The water-in-oil formulation of MA-OHE/ZnAC PE was prepared using the anti-solvent method [49]. Briefly, 1 g of cetyl of palmitate was mixed into 200 mL of glycerol, which was used to form the oil phase, and then heated to 60 °C. The oil phase was homogenized at 500 rpm for 1 min, and the speed was increased to 6000 rpm, following which 10 mL of the aqueous phase was added dropwise into the oil phase. High-speed homogenization continued for 3 min. Finally, a lotion-like MA-OHE/ZnAC PE was obtained after cooling under ambient conditions. The obtained MA-OHE/ZnAC PE was dialyzed and freeze-dried for further analysis.

### 3.3. Morphological Structure of MA-OHE/ZnAC PE

Scanning electron microscopy (SEM) and dynamic light scattering (DLS) were used to demonstrate the morphology of MA-OHE/ZnAC PE. SEM was used to determine the morphological structure of MA-OHE/ZnAC PE (JSM-7500F, Jeol Ltd., Musashino, Akishima, Tokyo, Japan) and a DLS instrument (ELS-8000, Otsuka Electronics Co., Ltd., Kirakata-shi, Osaka, Japan) was used to measure its particle size (Z-average) distribution and polydispersity index (PDI). Adequate MA-OHE/ZnAC PE was dispersed in DI water and carried into a quartz cell for measurement.

### 3.4. Bioactive Compound Entrapment Efficiency

The bioactive compound entrapment efficiencies of MA-OHE/ZnAC and MA-OHE/ZnAC PE were measured using total phenolic assay with slight modifications [50]. This method was conducted because MA-OHE contains various bioactive compounds, and the total entrapped bioactive compounds could be quantified using this assay. Briefly, 80 μL of MA-OHE, ZnAC, or MA-OHE/ZnAC was added to different wells of a 96-well plate, and then 20 μL of Folin-Denis solution was added to each well and mixed with the test compounds. Next, 100 μL 2% (*w*/*v*) of Na_2_CO_3_ solution was added to each well. The plate was incubated for 1 h in the dark at 22 °C, and absorbance was measured at 750 nm using a BioTek Synergy H1 hybrid multi-mode reader (Agilent Technologies Inc., Santa Clarita, CA, USA).

Entrapment efficiency was calculated using the following formula:

$$Encapsulation\;efficiency\left(\%\right)=\frac{{MA-OHE}_{Total}-{MA-OHE}_{Free}}{{MA-OHE}_{Total}}\times 100$$

where MA-OHE_Total_ indicates the total phenolic content of MA-OHE added for MA-OHE/ZnAC or MA-OHE/ZNAC PE, and MA-OHE_Free_ indicates the total phenolic content of free MA-OHE amount in MA-OHE/ZnAC or MA-OHE/ZnAC PE.

### 3.5. In Vitro Bioactive Compound Dissolution Kinetic Analysis

Bioactive compound dissolution kinetics of MA-OHE/ZnAC was determined using the dialysis method [51]. Briefly, PBS (pH 7.4) was used as the test medium and calibrated at 75 rpm and 37 °C. Next, 0.5 g of MA-OHE/ZnAC was dispersed in 20 mL of test medium in a dialysis tube, and the dissolution test was performed in 480 mL of test medium. The medium was collected after 6, 12, 24, and 48 h. As MA-OHE contains various bioactive compounds, the total amount of the released bioactive compounds was measured via total phenolic content assay. The dissolved total bioactive compounds were quantified using the same method and conditions as for measuring their entrapment efficiency, as described above.

### 3.6. In Vitro Drug Permeation Kinetics

The in vitro drug permeation kinetics were determined using a transdermal diffusion system (DHC-6TD, Logan Instruments Corp., Somerset, NJ, USA) and artificial human epidermis tissue (PB-M, Logan Instruments Corp.) [52]. Briefly, PBS was used as the media, and the test was performed at 600 rpm and 32 ± 0.5 °C. After 30 min of equilibration, MA-OHE/ZnAC PE was spread on the membrane, and the media was collected after 30 min, or 1, 2, 4, and 24 h. The permeated amount was quantified using a UV-vis spectrometer (Varian Cary 50, Agilent Technologies Inc., Santa Clara, CA, USA). To measure the UV-vis absorbance of MA- OHE/ZnAC PE, MA-OHE/ZnAC PE was dispersed in DI water and moved into the quartz cell. The diffusion coefficient and flux were calculated using Fick’s law.

### 3.7. Antimicrobial Susceptibility Test

#### 3.7.1. Bacteria Culture

*S. aureus* (ATCC 23235) was purchased from the American Type Culture Collection (ATCC; Manassas, VA, USA), and *C. acnes* (KCCM 42791) was purchased from the Korean Culture Center of Microorganisms (KCCM; Seodaemun-gu, Seoul, Republic of Korea). Tryptone was purchased from Condalab (Torrejón de Ardoz, Madrid, Spain). Bacto Yeast Extract was purchased from Thermo Fisher Scientific Inc. Sodium chloride was purchased from Sigma-Aldrich. Luria-Bertani (LB) media was prepared using 5 g of tryptone, 2.5 g of yeast extract, and 5 g of sodium chloride. For making LB-agar plates, 10 g of agar powder (Junsei Chemical Co., Ltd., Chuo-ku, Tokyo, Japan) was added to the medium. Reinforced clostridial medium (RCM) and AnaeroBag were purchased from KisanBio (Seocho-gu, Seoul, Republic of Korea).

*S. aureus* was cultured in LB medium at 37 °C, while *C. acnes* was cultured in RCM at 37 °C under anaerobic conditions achieved using an AnaeroBag. For downstream experiments, 1.5 × 10^8^ CFU/mL (0.5 Mcfarland) of bacteria in the growth phase was used.

#### 3.7.2. Determination of Minimum Inhibition Concentration (MIC)

MIC values were determined using the broth micro-dilution method [53]. Briefly, MA-OHE, ZnAC, and MA-OHE/ZnAC were dispersed in PBS and diluted to concentrations of 10, 7.5, 5, 2.5, 1, 0.75, 0.5, 0.25, 0.1, 0.075, 0.05, 0.025, and 0.01 mg/mL. Next, 100 μL of *C. acnes* or *S. aureus* in growth phase was seeded into a 96-well plate, followed by the addition of 100 μL of the different test samples to the wells. The plate was then incubated for 24 h, after which 100 μL of *C. acnes* from relevant wells was seeded on RCM agar media and incubated for 74 h. Likewise, 100 μL *S. aureus* from relevant wells was seeded on LB agar media and incubated for 24 h. The MIC values were determined by comparing the colony amount to the control.

### 3.8. In Vitro Cell Viability Test

#### 3.8.1. Cell Culture

Dulbecco’s modified eagle medium (DMEM), fetal bovine Serum (FBS), and penicillin/streptomycin (pen/strep) were purchased from Thermo Fisher Scientific Inc. Immortalized human skin keratinocyte cell line (HaCaT) was purchased from Cell Lines Service (Hohenzollernring, Köln, Germany). Water-soluble tetrazolium (WST) solution was purchased from DoGenBio (Guro-gu, Seoul, Republic of Korea).

HaCaT cells were cultured in DMEM supplemented with 10% FBS and 100 U/mL penicillin/streptomycin in an incubator set at 37 °C and 5% CO_2_ [54]. The culture medium was changed daily.

#### 3.8.2. WST Assay

WST assay was performed to evaluate cell viability when HaCaT cells were cultured in the presence of MA-OHE, ZnAC, or MA-OHE/ZnAC [55]. Briefly, HaCaT cells were seeded into a 96-well plate (0.5 × 10^4^ cells/well) and incubated at 37 °C and 5% CO_2_ for 24 h. Next, 10 μL of MA-OHE, ZnAC, or MA-OHE/ZnAC of various concentrations (100, 75, 50, 25, and 10 μg/mL) was added to the cells and incubated for 24 h. After incubation, the medium was rinsed three times. Next, 10 μL of WST solution was added into each well, and cells were further incubated for 2 h. Finally, cell viability was measured using a plate reader at 450 nm (Perkin Elmer Multimode Plate Reader Victor X5, Perkin Elmer, Middlesex, MA, USA).

### 3.9. Statistical Analysis

Analysis of variance was performed, and the coefficient of determination values (R^2^) were calculated using SPSS ver.25.0. The graphs were generated using Origin software version 2022b.

## 4. Conclusions

In this study, a Pickering emulsion system was formulated by loading MA-OHE onto ZnAC for the treatment of acne-inducing microbes. Morphological structures of MA-OHE/ZnAC PE were obtained using DLS and SEM images. MA-OHE/ZnAC exhibited strong antimicrobial activity without exerting any cytotoxic effects on HaCaT cells. Specifically, the antimicrobial activity of MA-OHE/ZnAC against *C. acnes* was greater compared to the use of ZnAC alone due to the extended-release characteristic of MA-OHE/ZnAC. The antimicrobial activity of MA-OHE/ZnAC against *S. aureus* was also strong enough to be comparable to antibiotics. The entrapment efficiencies of MA-OHE/ZnAC and MA-OHE/ZnAC PE were measured, and MA-OHE/ZnAC PE was found to have a high bioactive compound entrapment efficiency. Furthermore, rapid permeation of MA-OHE/ZnAC PE was observed under in vitro conditions. Remarkably, no lag time was observed during the in vitro drug release kinetics assay. It seems that the major advantages of the MA-OHE/ZnAC PE formula are rapid permeation and extended release with high entrapment efficiency. Thus, ZnAC possibly acts as a bioactive compound carrier and an antimicrobial agent. Hence, MA-OHE/ZnAC is a potential novel antimicrobial agent that can be used for treating acne-inducing microbes. Moreover, our study highlighted the advantages of using Pickering emulsion for fabricating the MA-OHE/ZnAC delivery system. With emerging interest in lipid nanoparticles, the current research reinforces their advantages.

## Figures and Tables

**Table 1:** Antioxidant plant extracts for treating acne-inducing microbes.

Medicinal Plants	Bacteria	Additional Effects	References
*Rosmarinus officinalis*	*P. acnes* *S. aureus* *Kocuria* sp.	Anti-depressant effect Immunomodulatory function	[11,12,13]
*Acacia nilotica*	*P. acnes* *Kocuria* sp. *B. subtilis*	Wounds healing effect Gastrointestinal-disorder treatment Anti-cancer	[14,15]
*Ocimum tenuiflorum*	*P. acnes* *Kocuria* sp. *B. subtilis*	Anti-depressant effect Anti-insecticide effect	[14,16,17]
Aloe vera	*S. aureus* *T. mentagrophytes*	Anti-inflammatory effect Anti-cancer effect	[18,19,20]
*Camellia sinensis*	*P. acnes*	Anti-inflammatory Anti-cariogenic	[21,22,23]
*Hemidesmus indicus*	*S. epidermis* *S. aureus*	Anti-thrombotic effect Renoprotective effect	[24,25,26,27]

**Table 2:** MIC values of MA-OHE/ZnAC PE.

Compounds	Microbes	MIC Values
MA-OHE	*S. aureus*	0.75 mg/mL
MA-OHE	*C. acnes*	7.5 mg/mL
ZnAC	*S. aureus*	0.1 mg/mL
ZnAC	*C. acnes*	0.05 mg/mL
MA-OHE/ZnAC	*S. aureus*	0.1 mg/mL
MA-OHE/ZnAC	*C. acnes*	0.025 mg/mL

## Data Availability

Data sharing is not applicable to this article.

## Supplementary Materials

The following supporting information can be downloaded at: https://www.mdpi.com/article/10.3390/ijms24119669/s1.
