# Supplementary material for: A Bioactive Compound-Loaded Zinc-Aminoclay Encapsulated, Pickering Emulsion System for Treating Acne-Inducing Microbes
Source: Int J Mol Sci. 2023 Jun 2;24(11):9669. doi: 10.3390/ijms24119669 (PMC10253637; doi:10.3390/ijms24119669)
Supplement: Supplementary file 1 [file ijms-24-09669-s001.zip › ijms-2391036-supplementary.pdf]

Bio-active compound entrapped zinc-aminoclay loaded W/O Pickering emulsion for acne-inducing microbial treatment

Seong-Hyeon Kim[1], In-Sun Bae[2], Hyun Uk Lee[3], Ju-Young Moon[4*], Young-Chul Lee[1*]

[1] Department of BioNano Technology, Gachon University, 1342 Seongnam-Daero, Sujeong-Gu, Seongnam-Si, Gyeonggi-do 13120, Korea; shaera@gachon.ac.kr (S.-H.K.), dreamdbs@gachon.ac.kr (Y.-C.L.)

[2] Swsonaki inc., Gwangyang frontier-valley 3rd, 30 Gaseok-Ro, Seo-Gu, Incheon, Korea; swsonaki@naver.com (I.-S.B.)

[3] Research center for Materials Analysis, Korea Basic Science Institute, Daejeon 34133, Korea; leeho@kbsi.re.kr (H.U.L.)

[4] Department of Beauty Design Management, Hansung University, 116 Samseongyoro-16gil, Seoul 02876, Korea; bora7033@naver.com (J.-Y.M.)

*Correspondence: bora7033@naver.com (J.-Y.M.); dreamdbs@gachon.ac.kr (Y.-C.L.)

Keywords: Pickering emulsion, acne, antimicrobial, extended release, Zinc-aminoclay (ZnAC) *Opuntia humifusa*

(SUPPLEMENTARY INFORMATION)

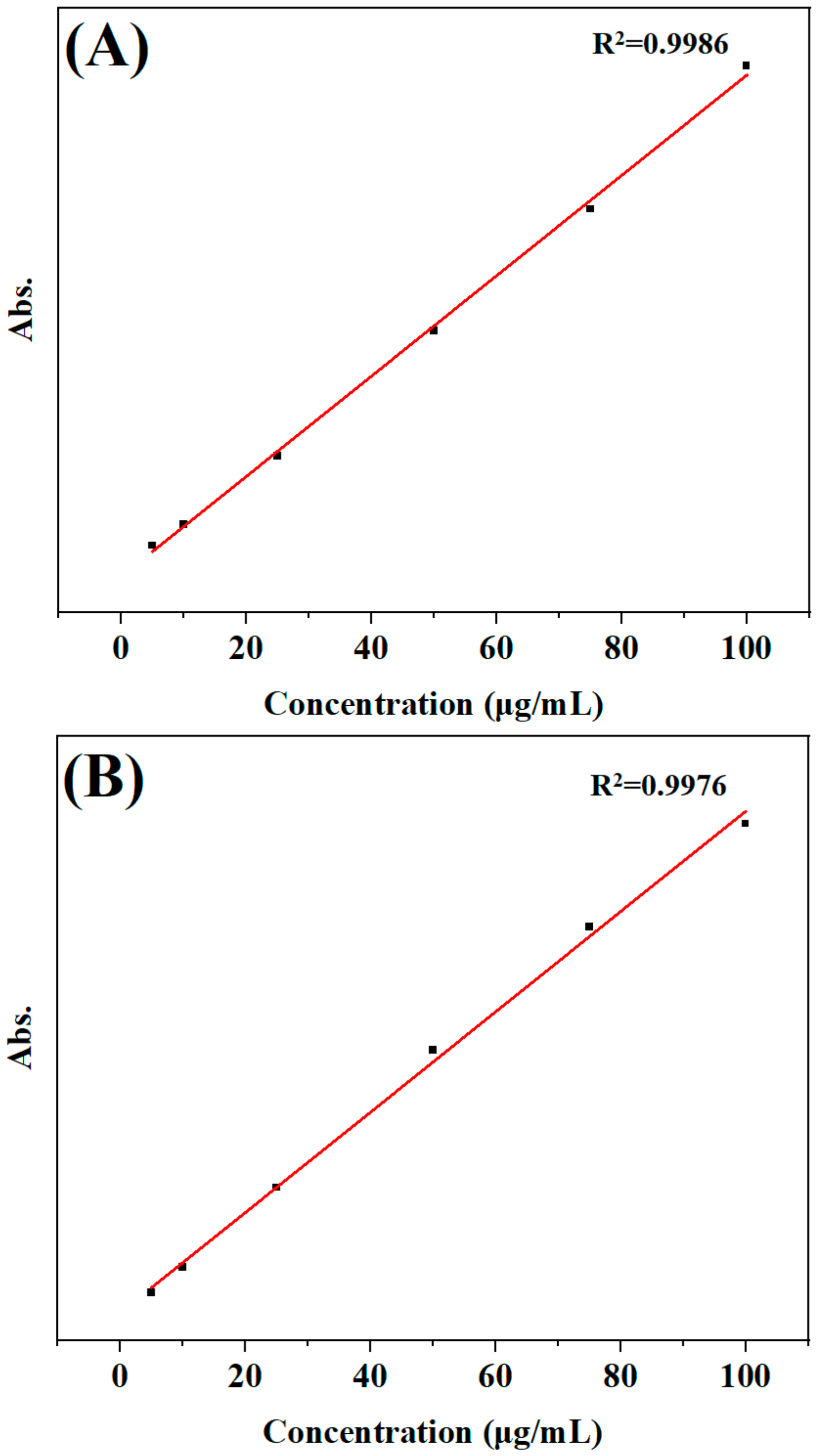

Figure S1. Standard curve of gallic acid for entrapment efficiency measurement of MA-OHE/ZnAC (A); and MA-OHE/ZnAC PE (B).

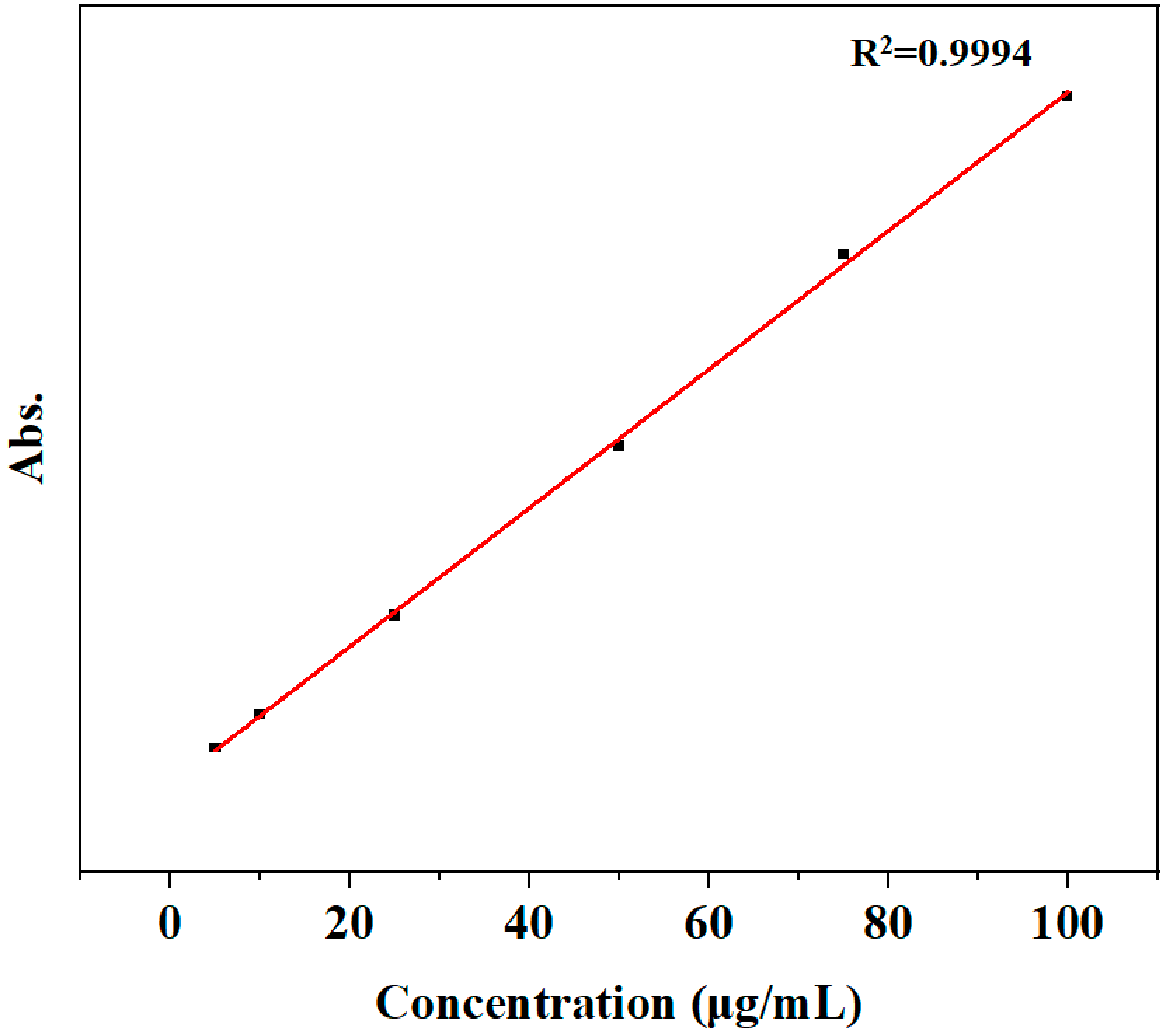

Figure S2. Standard curve of gallic acid for dissolution test of MA-OHE/ZnAC

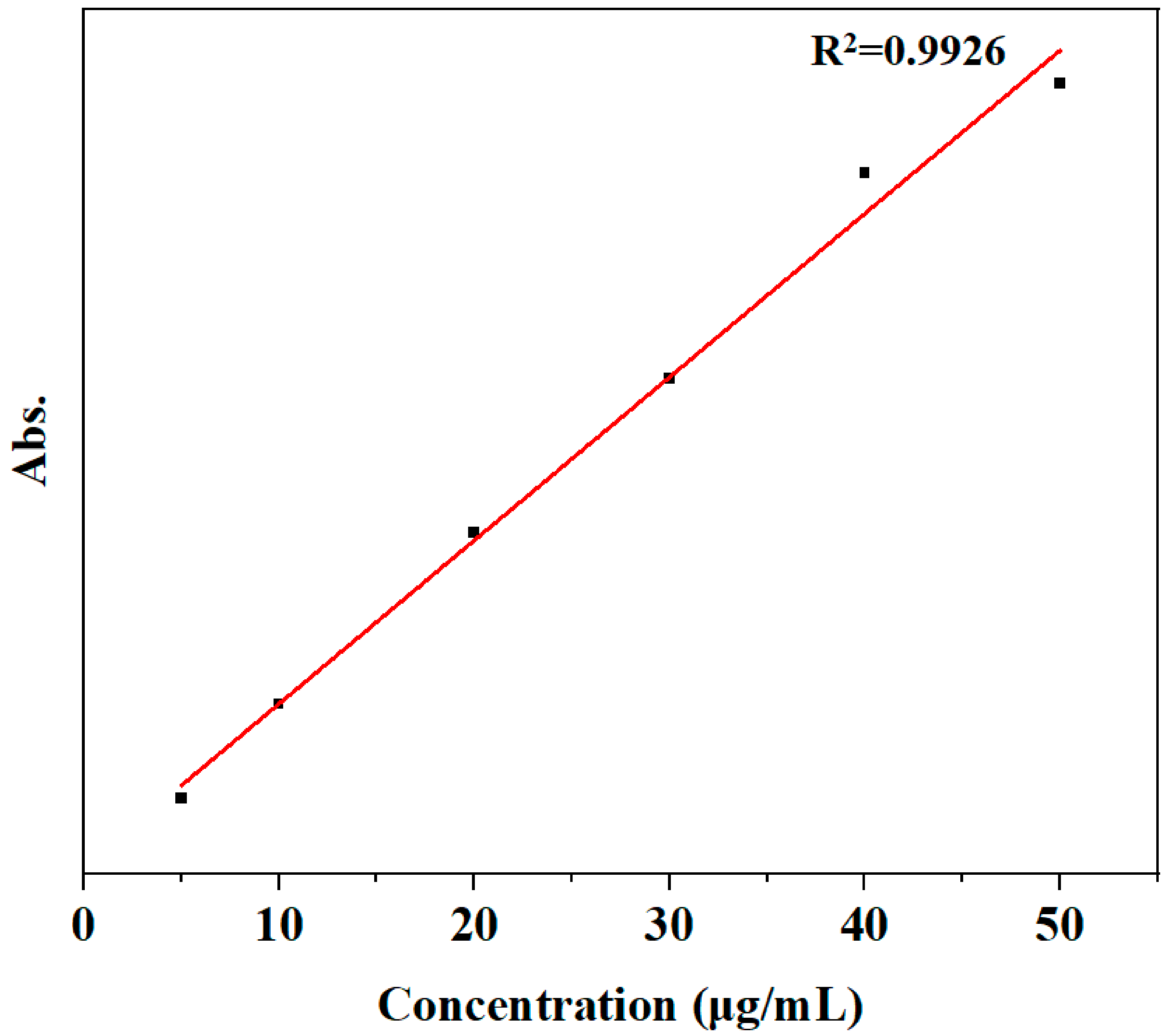

Figure S3. Standard curve of MA-OHE/ZnAC PE for *in vitro* drug release kinetics
